# A Role for Mental Imagery in the Experience and Reduction of Food Cravings

**DOI:** 10.3389/fpsyt.2014.00193

**Published:** 2015-01-06

**Authors:** Eva Kemps, Marika Tiggemann

**Affiliations:** ^1^School of Psychology, Flinders University, Adelaide, SA, Australia

**Keywords:** mental imagery, cravings, food, craving reduction, cognitive intervention, food intake

The term “craving” refers to a strong motivational state, which compels an individual to seek and ingest a particular substance (1). It usually refers to alcohol, tobacco, or drugs, but has become increasingly applied to food. Thus, food cravings refer to an intense desire or urge to eat a specific food (2). It is this specificity that distinguishes a craving from ordinary food choices and hunger (3). In Western societies, the most commonly craved foods are those high in fat, sugar, and salt, such as cake, chips, pizza, ice-cream, and in particular chocolate (4). Most people experience cravings for such palatable foods on occasion without any problem (5). However, food cravings can pose significant health risks for some people. Most notably, they can contribute to the development of obesity (6) and disordered eating (7), increasingly serious global health issues (8, 9). This has prompted a surge of investigations into the mechanisms that underlie the experience of food craving with a view to developing effective craving reduction techniques. The present paper focuses specifically on cravings for food, and the role of mental imagery in the experience and reduction of such cravings. For excellent reviews of the theoretical underpinnings of craving and addiction more broadly, we refer the reader to recent works by May and colleagues (10, 11). We take a more applied perspective here and critically evaluate the practical significance of imagery-based craving reduction interventions.

Over the past decade, a growing body of literature has highlighted a key role for mental imagery in the experience and reduction of food cravings. Experimental and survey data have shown that when people crave, they have vivid images of the desired food, including how delicious it looks and how good it tastes and smells (11–14). For example, when undergraduate students were asked to describe a previous food craving episode, 30% made explicit reference to mental imagery, using phrases such as “I could picture [the pizza] in my mind, picture eating it” (14). In addition, when presented with a list of descriptive statements, respondents strongly endorsed imagery-based descriptors as characteristic of their food cravings. Imagery descriptors in the visual (“I am visualizing the food”), gustatory (“I imagine the taste of the food”), and olfactory (“I imagine the smell of the food”) modalities in particular were rated highly; in contrast, auditory descriptors (“I imagine the sound of myself having it”) were not highly rated (12, 14). Furthermore, when asked to assign specific percentages to each of the five sensory modalities involved in an imagined food craving experience, the visual modality (39.7%) scored the highest, followed by the gustatory (30.6%) and olfactory (15.8%) modalities; by contrast, the tactile (9.5%) and auditory (4.4%) modalities were little used (14). These findings indicate that craving-related food images are predominantly visual, gustatory, and olfactory in nature.

Further evidence for the imaginal basis of food cravings comes from studies that have experimentally induced food cravings by instructing participants to imagine a food-related scenario (e.g., “Imagine you are eating your favorite food”) (15). Moreover, the strength of participants’ food cravings has been shown to correlate with the vividness of their appetitive images (16). In line with these empirical observations, a recent cognitive model of craving, the Elaborated Intrusion Theory of Desire (17), has placed vivid sensory images of the appetitive target at the very heart of the craving experience. According to this theory, sensory images are a key component of the cognitive elaboration that follows an initial intrusive thought about the craved substance.

More general cognitive psychological research has shown that the generation and maintenance of mental images (of whatever kind) can be disrupted by competing cognitive activities in the same sensory modality. For example, performing a visual task (e.g., watching a flickering pattern of black and white dots, termed dynamic visual noise) reduces the vividness of imagined objects or scenes, whereas engaging in a verbal task (e.g., counting aloud) reduces the vividness of imagined sounds (18). This occurs because of mutual competition between task performance and image maintenance for limited-capacity, modality-specific cognitive resources. Clinical applications of this dual-task methodology have shown that interference by a concurrent visual task can successfully reduce the vividness and emotional impact of distressing autobiographical images, characteristic of post-traumatic stress disorder (PTSD) (19–23).

Applications in the craving domain have similarly shown that competing cognitive tasks can disrupt desire-related mental images, and thereby can suppress cravings for alcohol, tobacco, and food. Notably, tasks that introduce competing information in the same sensory modality as the imagery associated with the craving, and thus compete for the same pool of limited-capacity resources, have proven the most effective. In particular, evidence from numerous laboratory studies has shown that engaging in a range of visual tasks can reduce food cravings. For example, imagining a series of non-food scenes (e.g., a rainbow) has been shown to reduce cravings for food in general (16) and for chocolate in particular (24). Other visual tasks, such as making hand or eye movements (25, 26), watching a dynamic visual noise array (25–29), constructing shapes from modeling clay (30), and playing a game of “Tetris” (31), have also been shown to reduce food cravings.

All these tasks are thought to have their craving reducing effect by reducing the vividness of visual craving-related images. Indeed, many of the previous studies not only assessed participants’ level of craving but also the vividness of their craving imagery, and reported a corresponding reduction in both measures under concurrent visual interference (25–31). However, in the absence of evidence for a causal relationship, the craving reducing effect could also reflect a reduction in the opportunity for craving imagery. In all this, it is important to note that people need not be good visualizers in general to derive benefit from imagery-based craving reduction tasks (26), thus demonstrating applicability across the board.

Although most research, to date, has focused on craving reduction via the visual sensory modality, there is evidence for a limited-capacity system that processes odor memory and imagery (32, 33) and is susceptible to olfactory interference (34, 35). Thus, the logic would suggest that a concurrent olfactory task would also reduce food cravings. In support, a number of studies have demonstrated craving reducing effects using olfactory tasks. The earliest such study showed that imagining the smell of non-food odors (e.g., eucalyptus, fresh paint) can reduce food cravings (24). More recent studies have shown that simply sniffing a non-food odorant, such as jasmine scented oil or a random chemical compound (36, 37), can also reduce food cravings.

In addition, a handful of studies has shown craving reducing effects from competing verbal tasks, such as imagining everyday sounds (e.g., a siren) (24), or listening to a foreign language recording (28). This suggests that competing tasks may act to distract participants or divert their attention. However, the craving reductions from verbal tasks in the above studies were substantially smaller than those produced by visual or olfactory tasks. Thus, although any cognitive task might serve as a distractor, it is tasks that engage the same cognitive processes as those used to construct and maintain craving-related imagery that will be most effective in reducing cravings. Further, although the great majority of people report craving-related images in the visual and olfactory/gustatory modalities (12), a minority do experience auditory images, and thus competing verbal tasks should reduce cravings for them.

All of the craving reduction studies described above have been conducted in the laboratory. These have induced food cravings experimentally using one of several methods, namely by depriving volunteers of food (30), instructing them to imagine eating a favorite food (16, 26, 27, 29), showing them pictures of food (25, 36, 37), or exposing them to actual food (24, 38). Recently, three studies have extended this laboratory work to the field. In the first, Knauper and colleagues (39) showed that a 4-day intervention whereby participants imagined themselves engaging in a favorite activity whenever they experienced a food craving reduced the intensity of naturally occurring cravings. Subsequently, Kemps and Tiggemann (40) showed that a concurrent visual task not only reduced everyday food cravings but also actual food intake. Specifically, they found that dynamic visual noise delivered on a hand-held electronic device reduced the strength of participants’ food cravings over a 2-week period, as well as the likelihood that they would eat in response to craving, and consequently the amount of calories they consumed. Most recently, Hsu and colleagues (41) showed that a 1-week evaluation of a mobile app that prompted participants to imagine a visual scene whenever they experienced a snack craving reduced snack consumption. These findings demonstrate the real-world applicability of imagery-based craving reduction techniques and in particular their utility for modifying craving-driven consumption.

One remaining limitation of the current research is that the majority of craving reduction studies in the food domain has been conducted with individuals of mostly normal weight. To date, only one study has demonstrated food craving reduction by an imagery-based technique in an overweight sample. Specifically, Kemps and colleagues (27) showed that dynamic visual noise was a more effective technique for reducing food cravings in overweight women on a prescribed weight-loss diet than was suppressing thoughts about food. This finding offers considerable scope for tackling unwanted food cravings, as experienced by individuals actively trying to lose weight (42), binge eaters (7), and some obese individuals (6).

Thus, converging evidence from numerous studies has shown that competing cognitive tasks that disrupt mental imagery can suppress food cravings. However, thus far research has shown only immediate craving reduction effects following the use of imagery-based techniques. This, of course, begs the question of the longevity of these effects. In the only study to date to investigate this question, Hamilton and colleagues (36) found that craving reduction effects from a guided imagery intervention were not sustained beyond the actual intervention. Craving levels after the intervention had reverted back to those observed at baseline. This suggests that competing cognitive tasks may disrupt craving imagery only temporarily, thereby providing momentary relief from the craving. Thus, imagery-based techniques may provide an effective “in-the-moment” tool for curbing food cravings, providing assistance in the “here and now.” Nevertheless, the field studies (39–41) suggest that imagery-based craving reduction techniques can be used successfully over the longer-term. While these techniques do not produce lasting reductions in craving, they do effectively reduce cravings on any one occasion. Moreover, their effectiveness does not diminish with repeated use. Indeed, the field studies clearly demonstrated that imagery-based techniques maintained their craving reducing effect with repeated use over several days (39), a week (41), and even over a couple of weeks (40).

Nevertheless, it is unlikely that imagery-based craving reduction techniques would be used as a stand-alone treatment. Although these techniques reliably reduce the craving, with reported reductions across studies around 20–25%, they do not eliminate it altogether. However, imagery-based techniques could be a useful adjunct to other therapeutic interventions for the treatment of craving-driven problematic eating behavior, for example, within the broader context of Cognitive Behavior Therapy. The Australian OnTrack program (http://www.ontrack.org.au) is an example of such an integrated treatment in the domain of alcohol dependence. Unlike other therapeutic techniques, imagery-based techniques involve very little effort on the part of the user. Visual and olfactory tasks also lend themselves for everyday use as a self-help tool to resist unwanted food cravings. Computerized visual tasks, such as dynamic visual noise and “Tetris,” could be easily incorporated as downloadable apps on smart phones and other hand-held devices. In fact, as noted above, Hsu and colleagues (41) recently designed an app to help users combat in-the-moment snack cravings (and consumption) by prompting them to imagine a visual scene. Additionally, commercially available non-food odorants can be purchased locally and carried around in people’s pockets and handbags. In this way, imagery-based craving reduction techniques can be readily accessible in a discreet manner virtually anywhere and anytime when a food craving arises.

In conclusion, increasing evidence highlights a key role for mental images in the experience of food cravings. Interference with these images from modality-specific cognitive tasks can effectively suppress such cravings, thereby paving the way for clinical interventions that target craving-driven problematic eating behavior.

## Conflict of Interest Statement

The authors declare that the research was conducted in the absence of any commercial or financial relationships that could be construed as a potential conflict of interest.
